# Molecular Investigation on Tick-Borne Hemoparasites and *Coxiella burnetii* in Dromedary Camels (*Camelus* *dromedarius*) in Al Dhafra Region of Abu Dhabi, UAE

**DOI:** 10.3390/ani11030666

**Published:** 2021-03-02

**Authors:** El Tigani Ahmed El Tigani-Asil, Valeria Blanda, Ghada Elderdiri Abdelwahab, Zulaikha Mohamed Al Hammadi, Shameem Habeeba, Abdelmalik Ibrahim Khalafalla, Mohamed Ali Alhosani, Francesco La Russa, Sergio Migliore, Alessandra Torina, Guido Ruggero Loria, Salama Suhail Al Muhairi

**Affiliations:** 1 Abu Dhabi Agriculture and Food Safety Authority (ADAFSA), Abu Dhabi Emirate 52150, United Arab Emirates; eltigani.mohammed@adafsa.gov.ae (E.T.A.E.T.-A.); Ghada.AbdelWahab@adafsa.gov.ae (G.E.A.); zulaikha.alhammadi@adafsa.gov.ae (Z.M.A.H.); shameem.beevi@adafsa.gov.ae (S.H.); abdelmalik.khalafalla@adafsa.gov.ae (A.I.K.); mohamed.a.alhosani@adafsa.gov.ae (M.A.A.); salama.almuhairi@adafsa.gov.ae (S.S.A.M.); 2 Istituto Zooprofilattico Sperimentale della Sicilia, Via Gino Marinuzzi 3, 90129 Palermo, Italy; francesco.larussa@izssicilia.it (F.L.R.); sergio.migliore@izssicilia.it (S.M.); alessandra.torina@izssicilia.it (A.T.); guidoruggero.loria@izssicilia.it (G.R.L.)

**Keywords:** dromedary camels, ticks, hemoparasites, molecular detection, phylogenetic analysis: United Arab Emirates

## Abstract

**Simple Summary:**

Dromedary camels (*Camelus dromedarius*), or Arabian camels, are mainly widespread in arid regions from the east of Asia to the north of Africa. Many species of parasites/arthropods affect camels, including ticks, able to transmit pathogens to animals and humans. Authors investigated the presence of blood parasites in *n* = 93 camels with acute clinical signs and in *n* = 72 ticks collected from these camels in Al Dhafra region of Abu Dhabi, United Arabian Emirates, through molecular techniques. All the 72 ticks collected were identified as *Hyalomma dromedarii* species and were found negative for pathogen DNA. DNA investigations on camel blood samples showed a positivity for tick-transmitted pathogens in 15 heads (16.1%): 15 *Anaplasma phagocytophilum* (11.8%), *Coxiella burnetii* (3.2%), and *Babesia*/*Theileria* spp. (2.1%). Coinfection of *A. phagocytophiulm* and *C. burnetii* was detected in a camel. *C. burnetii* sequences from our samples showed a high phylogenetic relatedness to strains from Europe, Africa, and Asia. The study represents the first molecular investigation on tick-borne pathogens in camels from United Arabian Emirates, and it highlights the possible risk of infection for humans working in close contact with camels.

**Abstract:**

Camels represent an important resource for inhabitants of the most arid regions of the world and their survival is mainly related to environment conditions including the risk of parasitic diseases, which may represent a significant cause of losses in livestock production of these areas. Camels may be parasitized by several hematophagous arthropods, which can be vectors of several diseases including zoonosis. This study aimed to investigate in dromedary camels and their ticks the importance of tick-borne hemoparasites that might be responsible for a recent and obscure morbidity of camels in Al Dhafra region of Abu Dhabi, UAE. Blood samples and ticks from 93 naturally infected camels belonging to 36 herds, affected by variable acute clinical syndromes lasting from 3 to 5 days, were analyzed through molecular techniques for specific DNA presence of different blood pathogens: *Anaplasma* *marginale*/*Anaplasma* *ovis*, *Anaplasma phagocytophilum*, *Coxiella burnetii,* *Babesia* spp., and *Theileria* spp. DNA. All the 72 ticks collected belonged to the *Hyalomma dromedarii* species and were negative for blood pathogens. *n* = 15 camels (16.1%) were found positive to the following tick-borne hemoparasites: *A. phagocytophilum* 11 (11.8%), *Coxiella burnetii* 3 (3.2%), and *Babesia/Theileria* spp. 2 (2.1%). One singular camel showed coinfection of *C. burnetii* and *A. phagocytophiulm*. Genetic profile of *C. burnetii* showed a high phylogenetic relatedness to European, Asian and African *C. burnetii* strains. This is the first laboratory investigation on tick-borne pathogens in camels in UAE, and the first report of *A. phagocytophilum* and *C. burnetii*. Moreover, since the detected pathogens are recognized pathogens for humans, this study highlights the zoonotic risk for humans working in camel husbandry.

## 1. Introduction

The dromedary camels (*Camelus dromedarius*), or Arabian camels, are mostly prevalent in the arid regions from the east of Asia to the north of Africa [1] with an overall population of about 30 million animals [2]. Camels may be parasitized by several hematophagous arthropods, affecting significantly milk and meat production or causing mortality. Ticks belonging to the genera *Rhipicephalus*, *Hyalomma*, *Dermacentor*, *Ixodes*, *Amblyomma*, *Argas*, *Otobius,* and *Ornithodoros* are often reported on camels [3]. Ticks are able to transmit several zoonotic and non-zoonotic parasitic, viral, and bacterial pathogens [4]. It has been reported that several zoonotic bacteria of public health importance have previously been detected in tick populations parasitizing camels and also in the blood of camels [5]. In addition, several other studies showed a variety of pathogens infecting camels including *Coxiella burnetii* [6], *Rickettsia* spp. (*R. aeschlimannii, R. africae,* and *R. sibirica mongolitimonae), Bartonella (B. bovis* and *B. rochalimae*), *Anaplasma phagocytophilum, Borrelia burgdorferi* (sensu lato) (s.l.) [7], and *Theileria* spp. (*T. dromedarii, T. camelensis,* and *T. annulata*) [8,9,10]. Studies in neighboring countries such as Iran and Saudi Arabia have reported *Theileria* spp., *Babesia* spp., and *Anaplasma* spp. in camels [11,12,13]. Incidence of clinical theileriosis in dromedary camels have been reported in the United Arab Emirates [14]. *T. annulata* antigen was detected in *Hyalomma dromedarii* ticks in the Eastern region of Abu Dhabi [15]. In several studies, sub-clinical anaplasmosis was reported in camels, and *A. camelii*, *A. ovis*, *A. platys,* and *A. phagocytophilum* were the most prevalent pathogens in both Dromedary and Bactrian camels [16,17,18,19]. Blood parasites, mainly *Theileria* and *Anaplasma* genera, significantly affect hematological and biochemical profile of animal hosts indicating that such pathogens interfere with the hepatic, renal, and muscular functions [20,21].

Published studies on camel pathogens have focused on case reports or studies of prevalence carried out by microscopical examination of fecal samples or blood smears, whereas identification of parasites by more specific molecular biology tools is less common [5]. However, new molecular methods for diagnosis of blood pathogens have provided data on prevalence of these parasites with far greater accuracy than conventional microscopy. Moreover, considering the difficulty of protozoa or rickettsia isolation even in the most advanced veterinary diagnostic laboratories, DNA detection has taken over as the “gold standard” for the diagnosis of parasitic infections [22].

There is lack of information on hemoparasites of camels in the Middle East and their significance on health and productivity. Arid climate, abundance of tick vectors, extensive movement of livestock between neighboring countries, and lack of a large-scale integrated tick control program make the region vulnerable to tick-borne diseases causing severe economic losses to camel owners and raises implications over the human health due to the zoonotic pathogens.

In this context, the current study is aimed to detect by molecular techniques the intracellular tick-borne hemoparasites circulating in sub-clinically infected dromedary camels and related ticks in the emirates of Abu Dhabi (Al Dhafra region).

## 2. Materials and Methods

### 2.1. Study Area

Al Dhafra is one of the three regions in the Emirates of Abu Dhabi, forming the Western part of the United Arab Emirates and it represents by far the largest region by area, occupying 71% of the Emirate’s total area. The Tropic of Cancer runs through the southern part of the Emirate, giving its climate an arid nature characterized by high temperatures throughout the year, and a very hot summer (June to August) associated with high relative humidity, especially in coastal areas (Supplementary FileSupplementary File, Figure S1).

### 2.2. Targeted Animals and Sample Collection

*N* = 93 clinically infected camels from *n* = 36 herds were selected for incidental investigation on tick-borne hemoparasites and other bacterial pathogens. Target animals were selected on observation of variable acute clinical signs including pyrexia, anorexia, swelling of external lymph nodes, edematous swellings, lacrimation, or nervous signs commonly observed in other ruminants infected with blood parasites. Blood samples were collected from jugular vein of 93 individual infected camels of 3 years and above ages, utilizing commercial vials (BD Vacutainer^®^, Bristol Circle Oakville, ON, Canada) containing EDTA and, before 24 h, taken to the Diagnostic Laboratories. Approximately, 1 mL blood was spotted on Whatman No. 4 filter paper, dried at room temperature, and stored at −20 °C for further DNA extraction and analysis. Blood cells were separated from the serum by centrifugation and 70 out of the 93 samples were also investigated for hematology and chemistry profile as previously described [23]. Ticks were randomly collected from the 93 camels reared in the study area and stored at room temperature in 70% ethanol.

### 2.3. Tick Species Identification

Ticks were identified under a stereomicroscope analyzing the external morphological characteristics using morphological keys reported in literature [24].

Molecular identification has been carried out as previously described [25,26]. Briefly, ticks were sectioned longitudinally and one half of each tick was used for DNA extraction using the Pure link Genomic DNA Kit (Thermo Fisher™ Applied Biosystems™, Waltham, MA, USA) according to the manufacturer’s instruction. The extracted DNA was quantified by Nanodrop 2000 (Thermo Fisher) and stored at −20 °C until use.

A fragment of 360 bp of the mitochondrial small subunit 12S rRNA gene was amplified by PCR using the primers T2A, 5*′*-AAATGAGAGCGACGGGCGATGT-3*′*, and T1, 5*′*AAACTAGGATTAGATACCCT 3*′* [27]. PCR was performed in a MJ Research PTC-200 Peltier Thermal Cycler (Hampton, NH, USA). Thermal profile included an initial denaturation at 95 °C for 15 min, followed by 35 cycles of denaturation at 95 °C for 30 s, annealing at 51 °C for 30 s, and elongation at 72 °C for 1 min. A final extension at 72 °C for 5 min was performed.

### 2.4. Extraction of DNA from Camels

DNA from dried blood spots on filter papers was extracted following method previously described [28]. Briefly, under aseptically condition, dried blood spots were cut into small pieces with sterile scissors and transferred into 1.5 mL microtubes containing 500 µL PBS; the mixtures were incubated at 37 °C for 15 min and then centrifuged at 8000 rpm for 3 min. Finally, 200 µL DNA was extracted using QIAamp DNA Mini Kit (QIAGEN, Hilden, Germany) following the manufacturer’s instructions. The extracted DNA was quantified by Nanodrop 2000 (Thermo Fisher) and stored at −20 °C until use.

### 2.5. Polymerase Chain Reaction

Nucleic acids extracted both from 93 camels and from 72 ticks were analyzed by PCR to detect DNA of *Anaplasma marginale/A. ovis* [29,30], *Anaplasma phagocytophilum* [31], and *Coxiella burnetii* [32]. Primers are tabulated in the Supplementary File (Table S1).

PCR was performed using GoTaq Polymerase (Promega, Madison, WI, USA). For each reaction, a positive control, consisting of an in-house prepared reference DNA obtained from positive field samples confirmed by sequencing, and a negative control were used. PCR was performed in a MJ Research PTC-200 Peltier Thermal Cycler. For each PCR, the thermal profiles are reported in the Supplementary File (Table S1). PCR products were visualized under UV after electrophoretic migration on 1.5% agarose gel containing SYBR Safe 1X (Invitrogen by © 2021 Life Technologies Corporation, Carlsbad, CA, USA).

### 2.6. Reverse Line Blot (RLB) for Babesia and Theileria spp.

*Babesia* and *Theileria* spp. DNA was detected by Reverse Line Blot (RLB). The hypervariable V4 region of the 18S rRNA gene of *Babesia* and *Theileria* species was amplified by PCR as previously described [33]. In particular, a touchdown PCR program was used in order to minimize nonspecific amplification. The protocol included 3 min at 94 °C, two cycles of 20 s at 94 °C, 30 s at 67 °C, and 30 s at 72 °C, and then two cycles with conditions identical to the previous cycles but with an annealing temperature of 65 °C. During subsequent two cycle sets, the annealing temperature was lowered by 2 °C until it reached 57 °C. Then, an additional 40 cycles each consisting of 20 s at 94 °C, 30 s at 57 °C, and 20 s at 72 °C were performed. The PCR was ended by an extra incubation for 7 min at 72 °C.

Reactions were carried out in 50 μL with 5 μL of DNA extracted from camel blood in a MJ Research PTC-200 Peltier Thermal Cycler. PCR products were then used for RLB hybridization, as described by [33,34,35,36]. Used oligonucleotide probes are reported in the Supplementary Files (Table S2). After hybridization, the membrane was exposed to a chemiluminescent detection film (GE Healthcare, Chicago, Illinois, USA) for 60 min to 24 h and then developed on Develop X-ray film (Agfa-Gevaert, Mortsel, Belgium) and fixed X-ray film (Agfa-Gevaert, Mortsel, Belgium). A black spot in the sample–probe cross in the hyper film indicated a positive signal for that pathogen.

### 2.7. Sequencing

A sequencing step was carried out for positive PCR reactions. PCR products for sequencing were purified using the Wizard SV Gel and PCR Clean-up System (Promega, Madison, WI, United States), quantified, and sent for sequencing (© Macrogen Europe, Amsterdam, The Netherland).

The obtained sequences were analyzed for nucleotide sequence identity by comparing them with reference strains in the GenBank database using the Basic Local Alignment Search Tool (BLAST, available online at https://blast.ncbi.nlm.nih.gov/Blast.cgi?PROGRAM=blastn&PAGE_TYPE=BlastSearch&LINK_LOC=blasthome, accessed date 20 July 2020.

### 2.8. Phylogenetic Analysis

Multiple sequence alignments were performed using BioEdit (Ibis Biosciences, Carlsbad, CA, USA) and ClustalW version 2.0.10 (www.ebi.ac.uk/clustalw, accessed date 3 October 2020). Phylogenetic trees were constructed by the Maximum Likelihood and the Neighbor-joining [37] methods. The evolutionary distances were computed using the Kimura 2-parameter [38] method, as implemented in the Mega 7 package [39] deleting all the gap sites. Since sequences of *Coxiella* endosymbionts have been previously documented in UAE in Argasid ticks collected from seabirds [40], we included *Coxiella* endosymbionts sequences to investigate their phylogenetic relationship. *Legionella pneumophila* was included as outgroups. GenBank accession numbers of sequences used for phylogenetic analysis are reported in Table 1.

### 2.9. Statistical Analysis

Statistical analysis was performed using chi-square function in R software, version 4.0.4 [41] and Bonferroni correction was applied. Prevalence differences between the tick-borne hemoparasites detected inside the sample was tested. Value of *p* < 0.016 was considered significant.

## 3. Results

### 3.1. Hematology Profile

A total of 70 out of 93 camel blood samples were subjected to complete blood count (CBC). The most relevant hematological findings were neutrophilia 78.6% (55 samples), lymphopenia 75.7% (53 samples), and monocytosis 35.7 % (25 samples). Moreover, 57 samples (81.4%) showed a marked increase in heart and muscle creatine kinase enzyme (CK), 60 samples (85.7%) showed high values of hepatic and muscular enzymes aspartate transaminase (AST) and lactate dehydrogenase (LDH), while liver enzyme gamma-glutamyl transferase (GGT) showed variable results such as for other standard camel hematology parameters.

### 3.2. Tick-Borne Pathogens

All the 72 ticks collected from camels, as confirmed by both morphological and molecular methods, belonged to the *Hyalomma dromedarii* species. All the ticks resulted negative for all the hemoparasites investigated in the present study.

Out of the 93 examined blood samples coming from dromedarian camels, *n* = 15 (16.1%) were found positive to tick-borne hemoparasites (Table 2), *n* = 11 (11.8%) were positive to *A. phagocytophilum*, and three camels were positive to *C. burnetii* (3.2%). Coinfection of *A. phagocytophiulm* and *C. burnetii* was also detected in one camel. All the samples were negative to *Anaplasma marginale/A. ovis*. Reverse Line Blot detected two camels positive to *Babesia* spp./*Theileria* spp. 2 (2.1%). Significant statistical difference between the three tick-borne hemoparasites detected (*A. phagocytophilum, C. burnetii,* and *Babesia/Theileria spp.*) was observed (*p* < 0.016).

Their DNA reacted with the probes of *Babesia* spp./*Theileria* spp., *Babesia* sp.1, *Theileria* sp. 1, and *Theileria* sp. 2 but with no other specie-specific probes in the membrane.

The products of PCRs for *A. phagocytophilum* and *Babesia* spp*./Theileria* spp. were too weak for visualization and did not allow to obtain sequences of good quality. Sequences obtained from the *C. burnetii*-positive samples were submitted to GenBank with the following accession numbers: MW057691, MW057692, and MW057693, respectively. Our sequences showed 100% identity among them and shared between 99.7% and 100% nucleotide sequence identities with sequences previously reported in GenBank (JQ346185 and MK335933.1, respectively).

### 3.3. Phylogenetic Analysis

**Figure 1:**
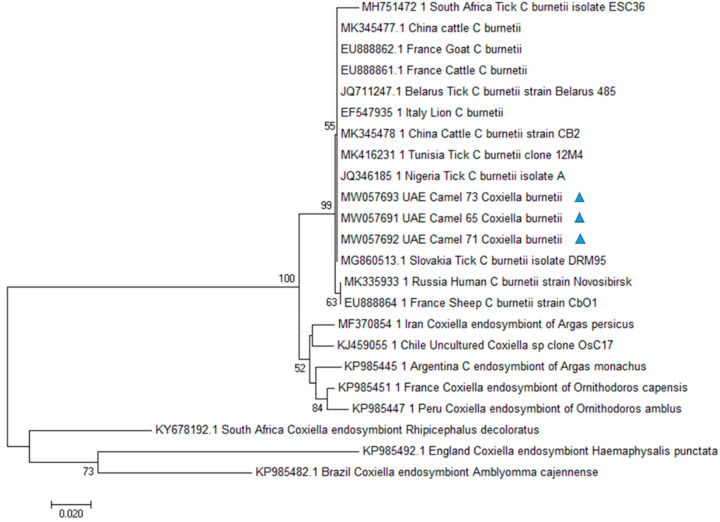
Phylogenetic analysis of *Coxiella burnetii* htpB gene sequences by Neighbor-Joining method based on the Kimura 2-parameter model. Evolutionary analyses were conducted in MEGA7. Numbers at nodes represent percentage occurrence in 1000 bootstrap replication. Sequences from this study are marked with triangle. The value lower than 50% are hidden.

*C. burnetii* obtained sequences were used for phylogenetic analysis (Figure 1). The inferred evolutionary history indicated that our sequences were classified in a single *C. burnetii* clade with sequences from Slovakia (MG860513.1), Belarus (JQ711247.1), Italy (EF547935.1), Nigeria (JQ346185.1), Tunisia (MK416231.1), and China (MK345478.1). Different clades were obtained with *Coxiella* endosymbiont sequences from different countries.

## 4. Discussion

Investigation of blood samples and ticks collected from naturally infected camels from different herds in Al Dhafra region of Abu Dhabi using molecular techniques enabled detection of DNA of tick-borne pathogens identified as *A. phagocytophilum, Coxiella burnetii,* and *Babesia/Theileria* spp. The main hematological and biochemical findings of this study were an increased WBC count as well as increase in heart, muscle, and liver enzymes indicating potential damage to cells of these organs. Similar profiles have been previously observed in camels suffering blood parasitic infections [20,21].

Data on identification of the tick population of Al Dhafra region confirmed previous studies on prevalence reporting that *Hy. dromedarii* is the main species parasitizing dromedary camels [42]. In this study, we did not find positive ticks to the examined pathogens, suggesting a limited risk of exposure through tick bites. Further studies are needed in order to get a more complete epidemiological picture of the area, enrolling more farms for a larger study of the camel population of UAE.

Nevertheless, the risk of health and welfare of the infested animals cannot be overlooked due to vector potential of *Hy. dromedarii* towards a high number of emerging and re-emerging pathogens, some of which also are zoonotic agents. Indeed, this tick species is considered the principal vector of *Theileria* spp. of domestic and wild ungulates in the Arabian Peninsula [42]. *Hy. dromedarii* has been confirmed to have a role in the transmission of *Rickettsia*, *Francisella*, and *C. burnetii* [43], and it is also a recognized vector of Crimean-Congo hemorrhagic fever virus [44].

The absence of detection of *Babesia* spp. and *Theileria* spp. DNA in the collected ticks is consistent with the low prevalence of these pathogens reported by previous studies carried out on tick-borne diseases of camels [15,42,45].

*Hy. dromedarii* could be also considered as potential vector in maintaining the endemism of *C. burnetii* in nature but not essential for its natural cycle and endemism maintenance in livestock.

Indeed, *C. burnetii* can be transmitted through inhalation of contaminated aerosols from amniotic fluid, placenta, or contaminated wool or by direct consumption of placentas or milk from infected animals [46].

To the best of our knowledge, this is the first report of *A. phagocytophilum* among naturally infected dromedaries in the study area. The result is even more important considering the zoonotic potential of this pathogen also because of its role as causative agent for human granulocytic anaplasmosis. Other reports of Anaplasmataceae in the *Camelus dromedarius* have been described elsewhere [47].

A study on *C. dromedarius* in Morocco, using PCR targeting the gene groEL and further sequence analysis of positive samples, revealed 100% identity with “*Candidatus* Anaplasma camelii.” Phylogenetic investigation and genetic characterization confirmed high similarity with *A. platys* [48]. *Anaplasma camelii* infection was also reported in healthy camels in Iran [16]. Dromedary camels were thought to be a reservoir for *Anaplasma phagocytophilum* as stated by Bahrami et al. [19]. Unidentified species belonging to family Anaplasmataceae were reported from EDTA whole-blood samples of dromedary camels, cattle, sheep, and desert foxes in Taif district, Kingdom of Saudi Arabia, when screened using PCR targeting 16S rRNA gene [12].

The present study reports for the first time the presence of *C. burnetii* DNA in camels in UAE. The htpB gene has previously been used for the detection of *Coxiella* organisms as well as for phylogenetic analyses [49,50]. HtpB sequences obtained in this study showed a high genetic relatedness with the ones of *C. burnetii* strains previously documented in Europe (Slovakia, Belarus Italy), Africa (Nigeria, Tunisia), and Asia (China), as supported by high bootstrap values. In UAE, *Coxiella burnetii*-like endosymbionts have been previously documented in Argasid ticks collected from seabirds [40]. Phylogenetic analysis carried out in that study showed that the reported *Coxiella*-like endosymbionts were in a separate cluster and not belonging to the *C. burnetii* group. In particular, the strain obtained from the argasid tick was closest neighbor to a *Coxiella* symbiont of *Ornithodoros (Carios) capensis*. A different molecular target, the isocitrate dehydrogenasegene, has been used for *Coxiella* sp. molecular characterization in that study, so that we could not include those sequences in our analysis. However, different htpB sequences from several endosymbiont *Coxiella* strains, including the sequence of a *Coxiella* endosymbiont of *Ornithodoros capensis*, have been processed in our phylogenetic analysis. Sequences from our samples were in a separate cluster from the above cited organisms, among the *C. burnetii* group and this topology of the Neighbor-Joining tree was supported by good bootstrap values.

This finding does not seem to support the same origin between the pathogen from ticks on seabirds and on camels. Further studies are thus required to better characterize the putative hosts and vectors of *C. burnetii* in UAE.

The detection of this pathogen DNA in three of the screened camels indicates the circulation of an abortion-causing pathogen that may have also implications on human health, being a zoonotic agent responsible for Q fever [51,52]. Several serological studies on *Coxiella burnetii* have been carried out in camels in several countries, reporting a seroprevalence of 40.7% in Egypt [53], 28.7% in Iran [54], 46% in Kenya [55], and 73% in Chad [56]. Studies based on molecular detection of *C. burnetii* DNA in camels are less frequent. A study on reproductive disorders in dromedary camel herds carried out in Saudi Arabia detected pathogen DNA by RT-PCR in 36% of uterine swabs collected from camels having a history of reproduction failure, representing the first association with reproductive disorders in dromedary camel herds [57].

The RLB analysis in this study reported two camels positive to *Babesia* spp. and *Theileria* spp. Their PCR products hybridized with the probes Cath all, *Babesia* sp.1, *Theileria* sp. 1, and *Theileria* sp. 2 and with no other specie-specific probe in the membrane, suggesting a different species from those present in the assay, although it was not possible to confirm this result with sequencing. Previous studies reported *Babesia* spp. DNA in the blood of 74.5% of the camels in the Eastern region of the Sudan [58]. An epidemiological study on the occurrence of blood parasitic infection in camels in Northern West Coastal zone of Egypt identified *Theileria*, *Anaplasma, Trypanosoma,* and *Babesia* by microscopy and PCR [22].

Microscopic examination of blood samples collected from camels in South of Iraq revealed the presence of *Babesia* spp. and *Theileria* spp. in camels at percentage of 9.95% and 5.8%, respectively. Molecular diagnosis based on PCR targeting 18S rRNA identified the pathogens as *B. caballi* and *T. equi* using specific primers [59]. A survey carried out in 200 peripheral blood samples from asymptomatic one-humped camels in Central and South-Eastern Iran, revealed one positive sample by PCR for *Theileria annulata* and *Trypanosoma evansi*. Sequence analysis confirmed 100% identity to *T. annulata* isolates from cattle [11]. Abdelwahab and colleagues [14] reported coinfection of salmonellosis and theileriosis in camels in UAE that clinically suffered from acute febrile disease with severe hematuria.

## 5. Conclusions

Camels represent a significant reservoir for zoonotic disease transmission to humans due to increased consumption and contact with camel meat and milk [60]. Our findings strongly support the need to carry out integrated strategy for animal diseases surveillance and prevention/control programs. In particular, the role of camels as hosts for zoonotic pathogens needs to be better investigated in order to understand the risk of zoonotic diseases, particularly, for humans working in close contact with camels or their products.

The major limitations of this study were the small number of samples collected and analyzed and the lack of good-quality sequences for *A. phagocytophilum* and *Babesia* spp./*Theileris* spp. Further studies involving a greater number of heads, herds, and ticks over a larger area of the UAE should be carried out in order to have a more accurate view of the prevalence of tick-borne pathogens and of the actual risk of disease for both animals and humans working in contact with camels in this country.

## Figures and Tables

**Table 1:** GenBank accession numbers of sequences used for phylogenetic analysis ^1^.

Organism	Host	Country	GenBank Accession Number
*C. burnetii*	*Homo sapiens*	Russia	MK335933.1
*C. burnetii*	*Amblyomma variegatum*	Nigeria	JQ346185.1 JQ346188
*C. burnetii*	*Hyalomma impeltatum*	Tunisia	MK416231.1
*C. burnetii*	Cattle	China	MK345478.1
*C. burnetii*	*Rhipicephalus evertsi evertsi*	South Africa	MH751472.1
*C. burnetii*	*Panthera leo*	Italy	EF547935.1
*C. burnetii*	*Ixodes ricinus*	Belarus	JQ711247.1
*C. burnetii*	*Dermacentor reticulatus*	Slovakia	MG860513.1
*C. burnetii*	Sheep	France	EU888864.1
*C. burnetii*	Cattle	France	EU888861.1
*C. burnetii*	Goat	France	EU888862.1
*Coxiella* endosymbiont of *Argas monachus*	*Argas monachus*	Argentina	KP985445.1
*Coxiella* endosymbiont of *Argas persicus*	*Argas persicus*	Iran	MF370854.1
*Coxiella* endosymbiont of *Ornithodoros capensis*	*Ornithodoros capensis*	France	KP985451.1
*Coxiella* endosymbiont of *Ornithodoros amblus*	*Ornithodoros amblus*	Peru	KP985447.1
*Coxiella* endosymbiont of *Haemaphysalis punctata*	*Haemaphysalis punctata*	England	KP985492.1
Uncultured *Coxiella* sp.	*Ornithodoros capensis* sensu lato	Chile	KJ459055.1
*Coxiella* endosymbiont of *Amblyomma cajennense*	*Amblyomma cajennense*	Brazil	KP985482.1
*Coxiella* endosymbiont of *Rhipicephalus decoloratus*	*Rhipicephalus decoloratus*	South Africa	KY678192.1

^1^ Date of accession to the GenBank database 2 October 2020.

**Table 2:** Results of molecular investigation on dromedary camels.

Investigation on Dromedarian Camels	Overall
Analyzed dromedarian camels	93
TBPs Prevalence	16.1% *
*Anaplasma marginale/A. ovis*	
Prevalence	0
*Anaplasma phagocytophilum*	
Prevalence	11.8%
*Coxiella burnetii*	
Prevalence	3.2%
*Babesia/Theileria* spp.	
Prevalence	2.1%

* Coinfection of *A. phagocytophiulm* and *C. burnetii* was detected in a camel.

## Data Availability

The data presented in this study are available on request from the corresponding author.

## Supplementary Materials

The following are available online at https://www.mdpi.com/2076-2615/11/3/666/s1, Table S1. PCR primers and amplification profiles. Table S2. Primers and Probes used for RLB targeting the V4 hypervariable region 18S rRNA of *Babesia* and *Theileria* species. Figure S1. Map of United Arab Emirates (UAE) showing in red color the location of Al Dhafra Region (the study area) in the Emirate of Abu Dhabi.
